# Optimizing Charging Efficiency and Maintaining Sensor Network Perpetually in Mobile Directional Charging

**DOI:** 10.3390/s19122657

**Published:** 2019-06-12

**Authors:** Xianghua Xu, Lu Chen, Zongmao Cheng

**Affiliations:** 1 School of Computer Science and Technology, Hangzhou Dianzi University, Hangzhou 310018, China; chenlu0154@hdu.edu.cn; 2 School of Science, Hangzhou Dianzi University, Hangzhou 310018, China; zmcheng@hdu.edu.cn

**Keywords:** wireless power transfer, directional charging vehicle, charging efficiency, RWSN

## Abstract

Wireless Power Transfer (WPT) is a promising technology to replenish energy of sensors in Rechargeable Wireless Sensor Networks (RWSN). In this paper, we investigate the mobile directional charging optimization problem in RWSN. Our problem is how to plan the moving path and charging direction of the Directional Charging Vehicle (DCV) in the 2D plane to replenish energy for RWSN. The objective is to optimize energy charging efficiency of the DCV while maintaining the sensor network working continuously. To the best of our knowledge, this is the first work to study the mobile directional charging problem in RWSN. We prove that the problem is NP-hard. Firstly, the coverage utility of the DCV’s directional charging is proposed. Then we design an approximation algorithm to determine the docking spots and their charging orientations while minimizing the number of the DCV’s docking spots and maximizing the charging coverage utility. Finally, we propose a moving path planning algorithm for the DCV’s mobile charging to optimize the DCV’s energy charging efficiency while ensuring the networks working continuously. We theoretically analyze the DCV’s charging service capability, and perform the comprehensive simulation experiments. The experiment results show the energy efficiency of the DCV is higher than the omnidirectional charging model in the sparse networks.

## 1. Introduction

Wireless power transfer is a promising technology to replenish energy to sensors in Rechargeable Wireless Sensor Networks (RWSN), to keep the network working continuously [1]. Wireless Power Transfer (WPT) is mainly using magnetic resonance coupling [1,2,3] or RF radiation technology [4,5]. To achieve efficient energy transfer in RF radiation technology, it generally requires directional transmission by using high-gain and directional antennas for power transmitters and receivers to focus the energy in narrow energy beams [6]. It has a more stable and higher efficiency of power transfer compared with omnidirectional charging [7]. Consequently, in the mobile directional charging scenario in RWSN, a rechargeable sensor can only receive power from a mobile charging vehicle equipped with a directional power transmitting antenna, or called directional charging vehicle (DCV), when they are located in the covered sector of the DCV’s directional antennas. 

Products from Powercast [8] carry out wireless charging by leveraging the electromagnetic radiation technique, with which energy transmitters broadcast the RF energy and receivers capture the energy and convert it to DC. Applications of the electromagnetic radiation technique for wireless charging have been reported in References [9,10,11]. As more and more applications of wireless charging technology have been envisioned, the Wireless Power Consortium [12] has been established to start the efforts of setting an international standard for interoperable wireless charging.

Recently, most research works of mobile charging in RWSN adopted the omnidirectional power transfer model [13,14,15,16,17]. Although some works have studied the directional charger’s deployment problem in RWSN [9,18,19,20], to the best of our knowledge, there is no literature that has studied the mobile directional charging problem. However, a directional antenna provides significant enhancement over the omnidirectional antenna in terms of direction beam [21]. Moreover, when charging distributed sensor nodes, a directional antenna, rather than an omnidirectional antenna, is more energy-efficient because of the smaller proportion of off-target radiation [22]. Inspired by the research issues in the literature on mobile omnidirectional wireless charging scheduling [13,14,15,16,17], and directional charger’s deployment [9,18,19,20], we propose the directional wireless charging optimization problem in this paper. The complex factors of RF power transmission in practical environment are simplified in our research problem.

**Figure 1:**
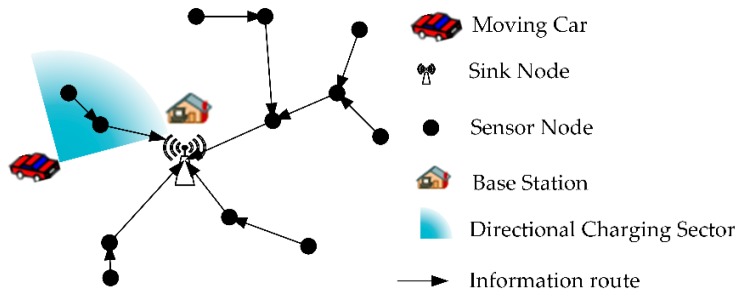
Directional mobile charging scenario for the data collection network in RWSN (Rechargeable Wireless Sensor Networks).

In this paper, we investigate the mobile directional charging optimization problem in wireless sensor networks. As shown in Figure 1, the data collection sensor network is deployed in a 2D plane area. The sensors transmit data to the sink node through multiple hops route. The charger’s base station serves for the DCV. The DCV starts from the base station and moves along the planned docking spots and path to replenishing energy for all sensors in a charging cycle. The mobile directional charging optimization problem is how to determine the DCV’s docking spots and charging directions in the 2D plane, and plan the moving path through all docking spots to replenish energy for the sensor network. The objective is to optimize Energy Charging Efficiency (ECE) of the DCV while maintaining the sensor network working continuously. The ECE is the ratio of the energy received by all sensors to the energy consumed by the DCV in a charging cycle. This problem is named as Charging Efficiency Optimization Problem (CEOP) of mobile directional charging in RWSN.

The CEOP problem has two main technical challenges. The first challenge is that since both the DCV’s docking spots and its charging orientations are continuous values, it is hard to determine the DCV’s docking spots and charging orientations to meet the charging coverage for all sensors. The second challenge is how to plan a DCV’s moving path that ensures no sensors will run out of energy during the charging cycle.

The CEOP is an NP-hard problem and it is difficult to design a global optimal solution. We consider dividing the CEOP problem into two sub-problems: (1) How to determine the appropriate docking spots of the DCV in the 2D plane and the DCV’s charging direction at each docking spot; (2) How to plan the DCV’s moving path and charging time at each docking spot to meet the network’s energy requirements and optimize the DCV’s energy charging efficiency.

We model the charging docking point planning on the 2D plane as a location optimization of mobile charging with the objective of minimizing the number of docking points under the constraints and of maximizing the charging coverage utility locally. Then, we use the TSP optimization to minimize the charging path loop and maximizing energy charging efficiency for the whole network.

The main contributions are as follow:

- As far as we know, this is the first work investigating the mobile directional charging problem in WRSN aiming to maximize the energy charging efficiency and maintain the networks working continuously.
- We prove that the problem is NP-hard.
- We propose the coverage utility of the DCV’s directional charging, and design an approximation algorithm to determine the docking spots and their charging orientations while minimizing the number of the DCV’s docking spots and maximizing the charging coverage utility. It ensures the mobile charging coverage for all the sensors in the network and improves the energy charging efficiency locally.
- We propose a moving path planning algorithm for the DCV’s mobile charging to optimize the DCV’s energy charging efficiency while ensuring the networks working continuously.
- We theoretically analyze the DCV’s charging service capability, and perform the comprehensive simulation experiments. The experiment results show that energy charging efficiency is higher than omnidirectional charging model in the data collection network.

The remainder of the paper is organized as follows: In Section 2, we review the related work of RWSN; In Section 3, we present the description of directional charging model, network energy consumption and problem definition; In Section 4, we propose the optimization algorithms; In Section 5, we give analysis of network size and area size that one DCV can serve; In Section 6, we present simulation result; Section 7 concludes this paper.

## 2. Related Works

The existing wireless energy transfer can be divided into Single-Input Single-Output energy transfer model [23,24,25,26,27,28,29,30,31,32,33,34] and Single-Input Multiple-Output energy transfer model [13,14,15,16,17,31,32,33,34,35]. Energy transfer optimization problems can be divided into static charging stations’ deployment [11,18,19,20,35,36,37,38] and mobile charging vehicles’ dispatching problems [13,14,15,16,17,23,24,25,26,27,28,29,30,31,32,33,34].

**Mobile omnidirectional wireless charging problem**. All existing works considering the mobile wireless charging adopt the omnidirectional power transfer model. Unlike the omnidirectional charging problem, we should not only determine the charging stop point and plan the charging path, but also determine the charging direction at each charging stop point. Yi et al. [13] investigate how to schedule the omnidirectional charging vehicle to maximize its vacation time and achieve higher charging efficiency of sensor networks. Xie et al. [17] investigate the mobile charging problem of co-locating the mobile base station on the wireless charging vehicle. Wu et al. [15] studied the omnidirectional charger vehicle dispatch problem to maximize the network lifetime and improve the energy efficiency for large-scale WSNs. Khelladi et al. [14] modeled the omnidirectional charger dispatching problem as a charging path optimization problem, and aimed to minimize the number of stop locations in the charging path and reducing the total energy consumption of the mobile charger. Jiang et al. [16] consider the on-demand mobile charging problem which schedules the omnidirectional charger to maximize the covering utility.

**Directional wireless chargers deployment problem**. All existing charging works which adopt the directional power transfer model only concern the directional chargers’ deployment problem in RWSN, rendering them not applicable to our problem. Dai et al. [9] investigated directional chargers’ deployment problem to optimize charging utility for the sensor network. Dai et al. [18] proposed the notion of omnidirectional charging and studied the omnidirectional chargeability under the deterministic deployment of chargers and random deployment of chargers. The goal is to achieve that at any position in the area with any orientation can be charged by directional chargers with power being no smaller than a given threshold. Jiang et al. [19] studied the wireless charger deployment optimization problem, which is to deploy as few as possible chargers to make the WRSN sustainable. Ji et al. [20] further investigated the deployment optimization problem of wireless chargers equipped with 3D beamforming directional antennas, and achieve the deployment of as few as possible chargers to make the WRSN sustainable. 

To best of our knowledge, this is the first work to study the mobile directional charging problem in RWSN. The closest to our work is mobile omnidirectional charging and deployment of directional charger. Compared with omnidirectional power transfer model, there are two strengths to introduce directional power transfer model in mobile charging application in RWSN. The first is that in the sparse sensor networks, using high gained RF radio directional power transfer antenna can reduce energy transmission waste and improve energy charging efficiency. The second is that the directional charger can cover longer distance and transfer more stable energy. 

## 3. Problem Formation

Table 1 describes the symbols used in this paper.

### 3.1. Directional Charging Model

**Figure 2:**
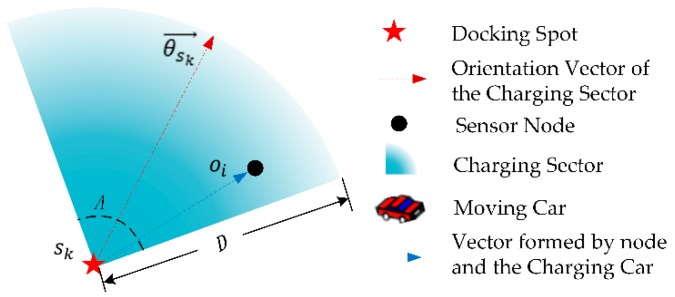
Directional Charging Model.

As shown in Figure 2, we introduce the DCV’s directional power transfer model as follows. When the effective charging distance of directional charger is D and charging coverage angle is A, the effective charging coverage area is a sector determined by its docking spot $s_{k}$ and charging orientation vector $\vec{\theta_{kj}}$. 

For a sensor node $o_{i}$ is located at $z_{o_{i}}$, in order to determine whether the node $o_{i}$ can be charged by the DCV stopped at docking spot $s_{k}$ with charging orientation vector $\vec{\theta_{s_{k}}}$, we have two judgment conditions: (1) The node $o_{i}$ is within the coverage angle A of the charger, denotes as inequality (1); and, (2) The distance between node $o_{i}$ and charger is less than D, denotes as inequality (2).

(1) $$\left(\;\vec{s_{k}-z_{o_{i}}}\right)\times \vec{\theta_{s_{k}}}\geq ∥s_{k}z_{o_{i}}∥\times cos\left(\frac{A}{2}\right)$$

where $∥s_{k}z_{o_{i}}∥$ denotes the distance between the location of the charger $s_{k}$ and the location of sensor node $z_{o_{i}}$.

(2) $$∥s_{k}z_{o_{i}}∥\leq D$$

We refer the RF wireless charging model in Reference [11] to calculate a node’s energy received from a wireless charger:

(3) $$P_{r}=\frac{G_{s}G_{r}\eta }{L}{\left(\frac{\lambda }{4\pi \left(d+\beta \right)}\right)}^{2}P_{out}$$

where d is the distance between a sensor node and a wireless charger, $P_{out}$ is the charger’s transmission power, $G_{s}$ is the transmitting antenna gain, $G_{r}$ is the node’s receiving antenna gain, L is polarization loss, λ is the wavelength, η is rectifier efficiency, and β is a parameter to adjust the Friis’ free space equation for short distance transmission. Except for distance d, all other parameters in Equation (3) are constant values based on the environment and device settings. Therefore, we simplify the charging model in Equation (3) as Equation (4).

(4) $$P_{r}=\frac{\alpha }{{\left(d+\beta \right)}^{2}}$$

where d is the distance from a sensor node to the DCV, and α represents other constant environmental parameters including $P_{out}$, $G_{s}$, $G_{r}$, L, λ and η in Equation (3). 

From Equation (4), we can deduce $P_{k,i}\left(s_{k},o_{i}\right)$, the effective charging power of the sensor node $o_{i}$ received from the DCV which stopped at docking spot $s_{k}$ with charging orientation vector $\vec{\theta_{s_{k}}}$:

(5) $$P_{k,i}\left(s_{k},o_{i}\right)=\{\begin{array}{c} \begin{array}{cc} \frac{\alpha }{{\left(d\left(s_{k},o_{i}\right)+\beta \right)}^{2}}, & \begin{array}{c} ∥s_{k}z_{o_{i}}∥\leq D\;and\\ \begin{array}{c} \left(\vec{s_{k}-z_{o_{i}}}\right)\times \vec{\theta_{s_{k}}}\geq \\ ∥s_{k}z_{o_{i}}∥\times cos\left(\frac{A}{2}\right) \end{array} \end{array} \end{array}\\ \begin{array}{cc} 0, & \;\;\;others \end{array} \end{array}$$

### 3.2. Network Energy Consumption Model 

We consider that each sensor node consumes energy for data sensing, transmission, and reception. We assume sensor node $o_{i}$ generates sensing data with a rate $R_{o_{i}}$(b/s). Assuming $PSN\left(o_{i}\right)$ is the set of previous sensor nodes that use sensor node $o_{i}$ on the routing path to the sink node. Equation (6) shows the total energy consumption of sensor node $o_{i}$.

(6) $$\omega_{o_{i}}=\sum_{o_{l\in PSN\left(o_{i}\right)}}\left(e^{t}+e^{r}\right)\times R_{o_{l}}+\left(e^{t}+\;e^{s}\right)\times R_{o_{i}}$$

Here $e^{s}$, $e^{t}$, and $e^{r}$ represent the energy consumption of one unit data for sensing, transmitting, and receiving respectively [15].

**Figure 3:**
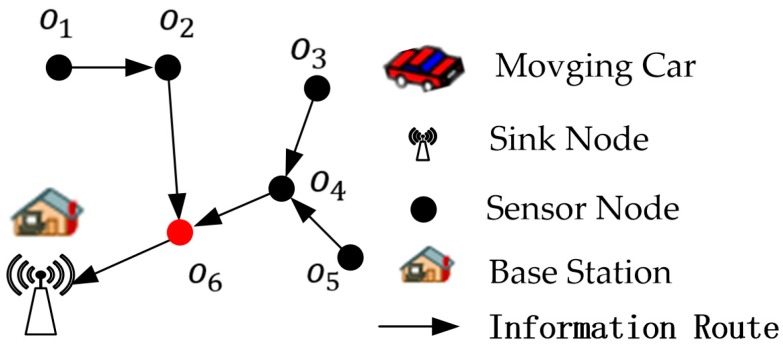
The data routing path to the Sink in the data collection network.

Then we determine the data routing of the network through the minimum energy routing [39]. As shown in Figure 3, nodes $o_{1}$,$\;o_{2}$,$\;o_{3}$,$\;o_{4}$, and $o_{5}$ sending data to the sink node through node $o_{6}$. Then, we have $PSN\left(o_{6}\right)=\left\{o_{1},o_{2},o_{3},o_{4},o_{5}\right\}$.

### 3.3. Problem Formulation 

**Figure 4:**
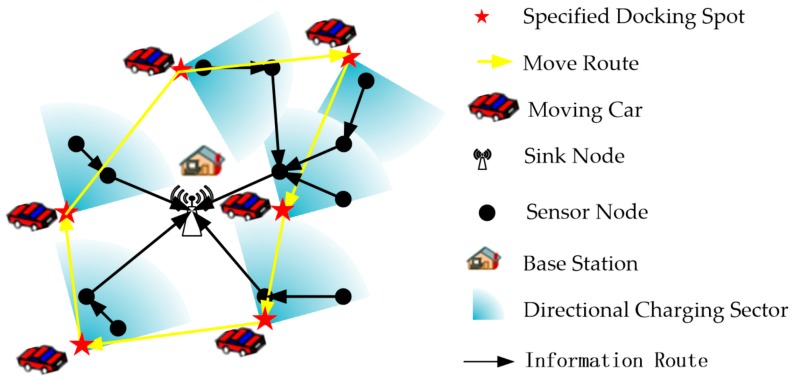
The DVC’s docking spots, charging orientations and moving path.

We consider a set of wireless rechargeable sensor nodes $O=\left\{o_{1},o_{2},\ldots ,o_{N}\right\}$ randomly distributed on a L×L 2D area, each sensor node $o_{i}$ generates sensing data with a rate $R_{o_{i}}$(b/s), i∈N. There is a sink node located at Base Station which gathers the data from all sensors in the sensor network. A Multi-hop data routing tree is constructed for forwarding all sensing data to the sink node, as shown in Figure 4. 

Aiming to keeping the network working continuously, a DCV with an energy capacity of $C_{max}$ is periodic dispatched to travel through a set of *Docking Spots* ($DS=\left\{s_{1},s_{2},\cdots ,s_{M}\right\}$), M denotes the number of docking spots. The DCV stops at each docking spot and rotates its RF charging antenna to a specific orientation to charging the nearby sensors.

In a charging cycle, the DCV starts from the base station, moves through each docking spot and finally returns back to the base station to wait for the next charging cycle. The charging cycle *T* consists of the moving time $T_{mov}$, the charging time $T_{cha}$, and the time rest at the base station $T_{res}$. The moving time $T_{mov}$ is determined by the length $L_{c}\;$of the DCV’s moving path and moving speed v. The charging time $T_{cha}$ is the sum of the dwell times at all docking spots, denote as $T_{cha}=\{t_{1}+\ldots +t_{k}+\cdots +t_{M}\}$. The remaining time of each cycle is the DCV’s rest time $T_{res}$.

(7) $$T=T_{res}+T_{mov}+T_{cha}=T_{res}+\frac{L_{c}}{v}+\overset{M}{\underset{k=1}{\sum }}t_{k}$$

Here Lcv denotes moving time of the DCV, $t_{k}$ denotes the DCV’s charging time at docking spot $s_{k}$, the sum of $t_{k}$ denotes the total charging time of the DCV.

Assume the DCV travels through DS to charging the sensor network. The DCV stops at a docking spot $s_{k}$ and rotates to a specific charging orientation vector$\;\vec{\theta_{s_{k}}^{l}}$. The sensor nodes which are effectively covered by the DCV denote as $SNC_{k}^{l}\left(s_{k},\vec{\theta_{s_{k}}^{l}}\right)$. The DCV’s dwell time is $t_{k}$ at docking spot $s_{k}$. 

For a DCV’s charging Path, $CP=BS⇢s_{p1}⇢\cdots s_{pk}⇢\cdots s_{pM}\},s_{pk}\in DS$, we define $E_{ECE}$, the *Effective Charging Energy* received by all sensor nodes from DCV in a charging cycle *T* as follows:

(8) $$E_{ECE}=\sum_{s_{k}\in DS}\sum_{o_{i}\in SNC_{k}^{l}\left(s_{k},\vec{\theta_{s_{k}}^{l}}\right)}P_{k,i}\left(s_{k},o_{i}\right)\times t_{k}$$

Here $P_{k,i}\left(s_{k},o_{i}\right)$ denotes the receiving power of the sensor node $o_{i}$ when the DCV is at docking spot $s_{k}$; and $SNC_{k}^{l}\left(s_{k},\vec{\theta_{s_{k}}^{l}}\right)$ denotes the sensor set covered by the DCV at $s_{k}$ and charging direction $\vec{\theta_{s_{k}}^{l}}$.

In a charging cycle *T*, the DCV’s energy consumption includes moving and charging energy, denote as $E_{mov}$ and $E_{cha}$ respectively. Charging consumption is determined by charging time and the DCV’s output power $P_{out}$. Moving energy consumption is determined by the length of path $L_{c}$ and its energy consumption per unit of moving length $\omega_{c}$. Then the DCV’s Energy Consumption, $E_{DCV}$ is denoted as Equation (9).

(9) $$E_{DCV}=E_{mov}+E_{cha}=P_{out}\overset{M}{\underset{k=1}{\sum }}t_{k}+\omega_{c}\times L_{c}$$

Here $t_{k}$ denotes the DCV’s charging time at docking spot $s_{k}$, $L_{c}$ denotes the DCV’s length of moving path, $\omega_{c}$ denotes DCV’s consumption power of moving.

We define the DCV’s Energy Charging Efficiency as follows.

**Energy Charging Efficiency**η: the ratio of effective charging energy received by the network to the DCV’s total energy consumption in a charging cycle T, denoted as Equation (10):

(10) η=EECEEDCV

Here $E_{ECE}$ denotes the *Effective Charging Energy* received by all sensor nodes from DCV in a charging cycle which can be calculated by Equation (8), and $E_{DCV}$ denotes the DCV’s energy consumption in a charging cycle which can be calculated by Equation (9).

We define the residual energy value of node $o_{i}$ at the time τ as $e_{o_{i}}\left(\tau \right)$ in a charging cycle. The node’s residual energy value at any time should be not lower than minimum value $E_{min}$, and not greater than maximum value $E_{max}$.

**Figure 5:**
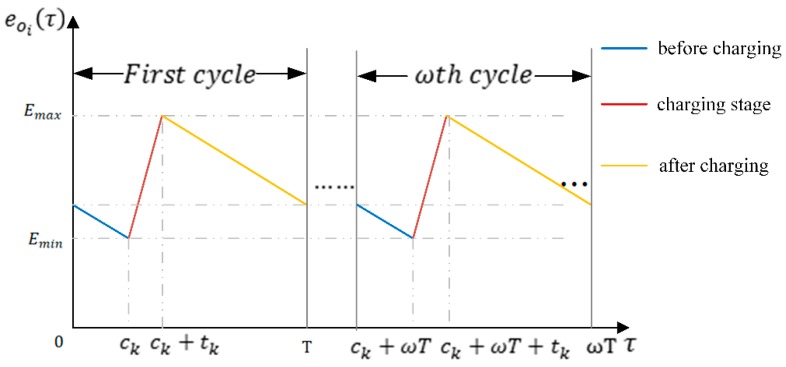
The variation of energy value function of node $o_{i}$.

The variation of node’s residual energy value in a cycle is divided into three stages: 1) before charging; 2) charging stage; 3) after charging. For a sensor node $o_{i}$, $e_{o_{i}}\left(\tau \right)$ varied in a charging cycle *T* as shown in Figure 5. $c_{k}$ denotes the arrival time of the DCV at docking spot $s_{k}$ in the first cycle *T*, $t_{k}$ denotes the DCV’s dwell time at docking spot $s_{k}$. 

The DCV carries limited energy $C_{max}$, so we have to make sure that the energy consumed by the charging car is no more than $C_{max}$ in a cycle. 

We define the CEOP problem of mobile directional charging as follow: 

For the set of wireless rechargeable sensor nodes $O=\left\{o_{1},o_{2},\ldots ,o_{N}\right\}$ randomly distributed on a L×L 2D area, how to plan the charging docking spots and charging path where the DCV moves along the path to replenishing energy for all sensors and maintains the sensor network working continuously. The objective is to maximize the DCV’s energy charging efficiency while maintaining the network working continuously.

**CEOP problem** is formulated as follow:

(11) maxη=EECEEDCVs.t.Emin≤eoi(τ)≤Emax,oi∈O,0≤τ≤ωTeoi(τ)={eoi(ωT)−ωoi×τ,τ∈[ωT,ωT+ck]Emin+(Pk,i(sk,oi)−ωoi)×τ,τ∈(ωT+ck,ωT+ck+tk]Emax−ωoi×τ,τ∈(ωT+ck+tk,(ω+1)T]EECE=∑sk∈DS∑oi∈SNC(sk,θskl→)Pk,i(sk,oi)×tkEDCV=Pout∑k=1Mtk+ωc×LcEDCV≤Cmax

Here η denotes Energy Charging Efficiency,$\;E_{max}$ denotes the maximum capacity of node, $E_{min}$ denotes minimum energy value of node. $e_{o_{i}}\left(\tau \right)$ denotes residual energy value of node $o_{i}$ at the time τ,when $\tau \in \left[\omega T,\omega T+c_{k}\right]$, $e_{o_{i}}\left(\tau \right)=e_{o_{i}}\left(\omega T\right)-\omega_{o_{i}}\times \tau$, $e_{o_{i}}\left(\tau \right)$ denotes the remaining energy of the node before charging, when $\tau \in (\omega T+c_{k},\omega T+c_{k}+t_{k}]$, $e_{o_{i}}\left(\tau \right)=E_{min}+\left(P_{k,i}\left(s_{k},o_{i}\right)-\omega_{o_{i}}\right)\times \tau$, $e_{o_{i}}\left(\tau \right)$ denotes the remaining energy of the node during charging, when $\tau \in (\omega T+c_{k}+t_{k},\left(\omega +1\right)T]$, $e_{o_{i}}\left(\tau \right)=E_{max}-\omega_{o_{i}}\times \tau$, $e_{o_{i}}\left(\tau \right)$ denotes the remaining energy of the node after charging. $E_{ECE}$ denotes the Effective Charging Energy received by all sensor nodes from the DCV in a charging cycle, $E_{DCV}$ denotes the DCV’s energy consumption in a charging cycle, $C_{max}$ denotes maximum energy capacity of the DCV.

## 4. Design and Analysis of Algorithms

It is difficult for the CEOP problem to be solved directly. We solve the problem in two steps and divide it into two sub-problems:

(1) First, we find the set of Docking Spots ($DS=\left\{s_{1},s_{2},\cdots ,s_{M}\right\}$) and their corresponding Charging Orientation ($CO=\left\{\vec{\theta_{s_{1}}},\vec{\theta_{s_{2}}},\cdots ,\vec{\theta_{s_{M}}}\right\}$) to maximize the charging coverage utility and ensure the mobile charging coverage of the network (Section 3.1).
(2) Second, we plan the DCV’s charging path to travel through all docking spot in DS and the charging residence time at each docking spot to optimize the overall energy charging efficiency while maintaining the sensor network working continuously (Section 3.2).

### 4.1. Find Charging Docking Spots and Charging Directions

For the 2D plane on which the sensors are randomly deployed, we divide it into grids, and take grid vertices as the DCV’s possible docking spots. Then we find the minimum number of the DCV’s candidate docking spots and their charging directions to optimize the charging coverage utility locally while achieving mobile charging coverage for the whole network.

We define the DCV’s Charging Coverage Utility at docking spot $s_{k}$ on the charging orientation $\vec{\theta_{s_{k}}^{l}}$ as the sum of received power of the charging covered nodes:

(12) $$U\left(s_{k},\vec{\theta_{s_{k}}^{l}}\right)=\sum_{o_{i}\in SNC\left(s_{k},\vec{\theta_{s_{k}}^{l}}\right)}P_{k,i}\left(s_{k},o_{i}\right)$$

where $SNC\left(s_{k},\vec{\theta_{s_{k}}^{l}}\right)$ denotes the sensor nodes covered at docking spot $s_{k}$ in charging orientation $\vec{\theta_{s_{k}}^{l}}$. 

Suppose at the docking spot $s_{k}$, the DCV has $Q_{k}$ optional charging directions, i.e., {$\vec{\theta_{s_{k}}^{1}}$,$\vec{\;\theta_{s_{k}}^{2}}$,…$,\;\vec{\theta_{s_{k}}^{Q_{k}}}$}. The maximum charging coverage utility at docking spot $s_{k}$ is $U_{max}\left(s_{k}\right)$:

(13) $$U_{max}\left(s_{k}\right)=max\left\{U\left(s_{k},\vec{\theta_{s_{k}}^{1}}\right),U\left(s_{k},\vec{\theta_{s_{k}}^{2}}\right)\cdots U\left(s_{k},\vec{\theta_{s_{k}}^{Q_{k}}}\right)\right\}$$

Here $U\left(s_{k},\vec{\theta_{s_{k}}^{Q_{k}}}\right)$ denotes charging coverage utility at docking spot $s_{k}$ in charging orientation $\vec{\theta_{s_{k}}^{l}}$.

For num grid points on the discrete 2D plane, we get the vertex set of grids: $CS=\left\{cds_{1},\cdots ,cds_{k},\cdots ,cds_{num}\right\}$,$\;cds_{k}$ is coordinates of vertexes. We have to choose a set of candidate docking spots $S=\left\{s_{1},\cdots ,s_{k},\cdots ,s_{M}\right\},\;s_{k}\in CS$, and their corresponding charging direction $\theta =\left\{\theta_{s_{1}},\cdots ,\theta_{s_{k}},\cdots ,\theta_{s_{M}}\right\},\;\theta_{s_{k}}\in \left\{\theta_{s_{k}}^{1},\ldots ,\theta_{s_{k}}^{Q_{k}}\right\}$, where $\;s_{k}$ has $Q_{k}$ possible charging directions. We use $U_{sum}^{max}\left(s\right)$ denotes the maximum coverage utility of the set *S* of candidate docking spots as Equation (14).

(14) $$U_{sum}^{max}\left(S\right)=\overset{M}{\underset{k=1}{\sum }}U_{max}\left(s_{k}\right)\;S=\left\{s_{1},\cdots ,s_{k},\cdots ,s_{M}\right\},\;s_{k}\in CS\;\theta =\left\{\theta_{s_{1}},\cdots ,\theta_{s_{k}},\cdots ,\theta_{s_{M}}\right\},\;\theta_{s_{k}}\in \left\{\theta_{s_{k}}^{1},\ldots ,\theta_{s_{k}}^{Q_{k}}\right\}$$

**Figure 6:**
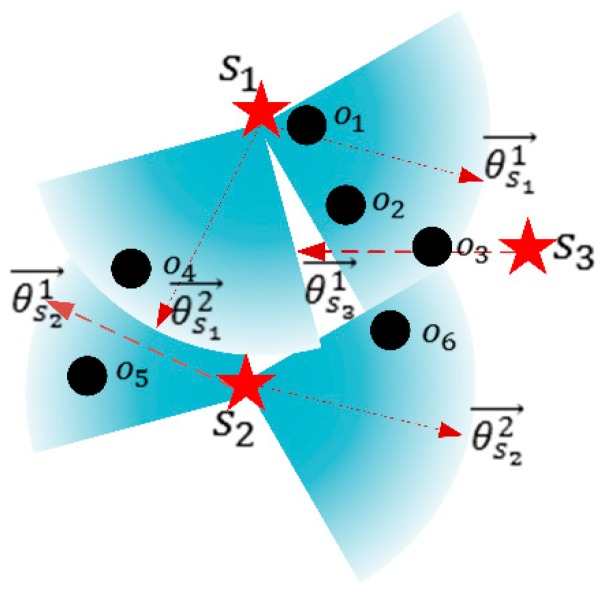
Combination of candidate docking spots and orientations.

As shown in Figure 6, there are three candidate docking spots $s_{1}$, $s_{2}$, and $s_{3}$. The docking spot $s_{1}$ can choose two possible orientation {$\vec{\theta_{s_{1}}^{1}}$, $\vec{\theta_{s_{1}}^{2}}$}, the docking spot $s_{2}$ can choose two possible orientation {$\vec{\theta_{s_{2}}^{1}}$, $\vec{\theta_{s_{2}}^{2}}$}, and the docking spot $s_{3}$ can choose orientation {$\vec{\theta_{s_{3}}^{1}}$}. Therefore, there are five different coverage utility of different combinations of docking spots and orientation vectors. We can calculate the possible coverage utilities at $s_{1}$, $s_{2}$, and $s_{3}$ according to Equation (12). For $s_{1}$, two possible coverage utilities are presented as Equation (15). For $s_{2}$, two possible coverage utilities are presented as Equation (16). For $s_{3}$, one possible coverage utility is presented as Equation (17).

(15) $$\{\begin{array}{c} U\left(s_{1},\vec{\theta_{s_{1}}^{2}}\right)=P_{1,4}\left(s_{1},o_{4}\right)\\ U\left(s_{1},\vec{\theta_{s_{1}}^{1}}\right)=P_{1,1}\left(s_{1},o_{1}\right)+P_{1,1}\left(s_{1},o_{2}\right) \end{array}$$

(16) $$\{\begin{array}{c} U\left(s_{2},\vec{\theta_{s_{2}}^{1}}\right)=P_{2,1}\left(s_{2},o_{4}\right)+P_{2,5}\left(s_{2},o_{5}\right)\\ U\left(s_{2},\vec{\theta_{s_{2}}^{2}}\right)=P_{1,6}\left(s_{2},o_{6}\right) \end{array}$$

(17) $$U\left(s_{3},\vec{\theta_{s_{3}}^{1}}\right)=P_{3,2}\left(s_{3},o_{2}\right)+P_{3,3}\left(s_{3},o_{3}\right)+P_{3,6}\left(s_{3},o_{6}\right)$$

As shown in Figure 6, we can get $U_{max}\left(s_{1}\right)$ and $U_{max}\left(s_{2}\right)$ respectively, as Equations (18) and (19).

(18) $$U_{max}\left(s_{1}\right)=U\left(s_{1},\vec{\theta_{s_{1}}^{1}}\right)$$

(19) $$U_{max}\left(s_{2}\right)=U\left(s_{2},\vec{\theta_{s_{2}}^{1}}\right)$$

Then we get the maximum coverage utility of candidate set $S=\left\{s_{1},s_{2},s_{3}\right\}$ and their related charging directions θ ={$\theta_{s_{1}}^{1},\theta_{s_{2}}^{1},\theta_{s_{3}}^{1}$}, $U_{sum}^{max}\left(S\right)=\;U\left(s_{1},\vec{\theta_{s_{1}}^{1}}\right)+U\left(s_{2},\vec{\theta_{s_{2}}^{1}}\right)+U\left(s_{3},\vec{\theta_{s_{3}}^{1}}\right)$.

The DCV’s energy loss includes charging energy loss and moving energy costs. At each docking spot, we aim to reduce the DCV’s charging loss and get higher charging effectiveness. By minimizing the number of docking spots, we can reduce the DCV’s moving energy cost in the process of mobile charging. Additionally, maximizing charging coverage utility can reduce the charging energy loss at each docking spot. Hence it finally improves the energy charging efficiency in mobile directional charging. 

To find the candidate docking spots and their charging directions for improvement of the mobile charging energy efficiency, we propose the two-objective optimization problem as Equation (20), that is Minimizing the number of Stop points and Maximizing charging Coverage Utility under the constraint of charging coverage of all sensors, called the MSMCU (Minimizing the number of Stop points and Maximizing charging Coverage Utility) problem.

(20) $$\begin{array}{cc} min & \overset{num}{\underset{k=1}{\sum }}a_{k}\\ max & \overset{num}{\underset{k=1}{\sum }}U\left(s_{k},\vec{\theta_{s_{k}}}\right)\\ s.t. & \overset{num}{\underset{k=1}{\sum }}a_{k}\times x_{i,k}\geq 1,\;1\leq i\leq N \end{array}\;x_{i,k}=\{\begin{array}{c} 1,if\;o_{i}\in SNC\left(s_{k},\vec{\theta_{s_{k}}^{l}}\right)\\ 0,if\;o_{i}\notin SNC\left(s_{k},\vec{\theta_{s_{k}}^{l}}\right) \end{array}$$

where $a_{k}$ is a binary decision variable that is equal to 1 if region $s_{k}$ belongs to the minimum stops, and to 0 otherwise. Additionally, the n inequality constraints ensure that every node must belong to at least one stop region in the minimum stops. We analysis Equation (20), give Theorem 1 and the proof of Theorem 1.

**Theorem** **1.** *The MSMCU problem of finding specified docking spots and orientations with minimum the number of stops and maximum coverage utility is NP-hard.*

**Proof of Theorem** **1.** We prove Theorem 1 by giving a special instance of the problem and explaining that the instance is NP-hard.

Instance. We assume that the coverage utility is the maximum as long as a sensor is covered, then the problem can be reduced to solve the Minimum Set Covering Problem. Because the Minimum Set Covering Problem is NP-hard, the MSMCU problem is also NP-hard. □

Then we propose a Greedy approximation algorithm of Maximum Coverage Utility (GMCU). GMCU algorithm firstly divides a 2D plane into grids. Secondly, it takes each grid vertex as a possible stop point and computes its optimal charging Direction and Maximum Coverage Utility (DMCU). Finally, it selects a set of candidate stop points to achieve overall maximum utility and network charging coverage. Let us first introduce the GMCU algorithm, and then introduce the DMCU algorithm.

**(1) GMCU algorithm**

In the GMCU algorithm, we divide the plane into grids, and take each vertex as a possible docking spot. The coverage of the charger is a 90° sector with radius D. The DCV only chooses one orientation to charge each time it stops, so if the grid’s size is too large, some nodes will be missed. The grid’s size d must satisfy Equation (21)

(21) $$d\leq \sqrt{2}/2\times D$$

The GMCU algorithm firstly divides a 2D plane into grids, take each grid vertex as a possible docking spot, denoted as CS, and $cds_{i}$ represents coordinates of vertexes. Put each $cds_{i}$ into the DMCU algorithm to calculate the maximum coverage utility and the covered nodes set. Choose the docking stops with the maximum value of coverage utility until all nodes are covered. The outputs are the docking spot set (DSS) and the set of covered nodes set (SANC) at corresponding directions.

The procedure of GMCU algorithm is presented in Table 2.

**(2) The DMCU algorithm**

The DMCU algorithm is used to find the charging direction with maximum coverage utility at each docking spot. 

**Figure 7:**
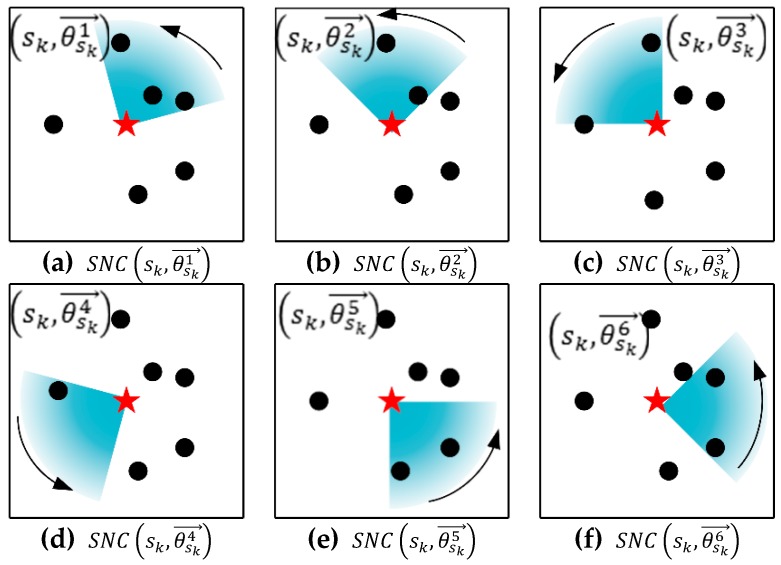
An example for showing the procedure of DMCU (Direction and Maximum Coverage Utility) algorithm.

Take Figure 7 as an example to illustrate the process of DMCU algorithm: (1) The DCV rotates counter-clockwise with each different node as initial boundary; (2) Calculate the coverage utility of each orientation. 

Six different coverage utility values can be obtained; the output is orientation with maximum coverage utility of a docking spot and the sensor nodes set that the combination of docking spot and this charging orientation can cover. Figure 7a–f show the set of nodes covered by the DCV at dock spot $s_{k}$ in each orientations ($\vec{\theta_{s_{k}}^{1}}\sim \vec{\theta_{s_{k}}^{6}}$), $SNC\left(s_{k},\vec{\theta_{s_{k}}^{1}}\right)\sim SNC\left(s_{k},\vec{\theta_{s_{k}}^{6}}\right)$ represent the corresponding nodes sets.

The procedure of DMCU algorithm is presented in Table 3.

*OCS* represents a coverage set of a candidate docking spot, the initial value is null. If the distance between the node and the candidate stop is not greater than D, then add the node into *OCS*. $\gamma_{j}$ in φ represents the angle formed by each node in OCS at each candidate dock spot. $DCS_{k}$ represents a coverage set of candidate stop with kth charging direction, and the $CUS_{j}$ indicates the corresponding coverage utility value. The DMCU algorithm finally outputs the maximum value $U_{s_{k}}^{max}\;$in $CUS_{j}$ and the set of covered nodes $SNC\left(s_{k},\vec{\theta_{s_{k}}^{l}}\right)$ covered at this docking spot $s_{k}$ with corresponding direction $\vec{\theta_{s_{k}}^{l}}$.

**Figure 8:**
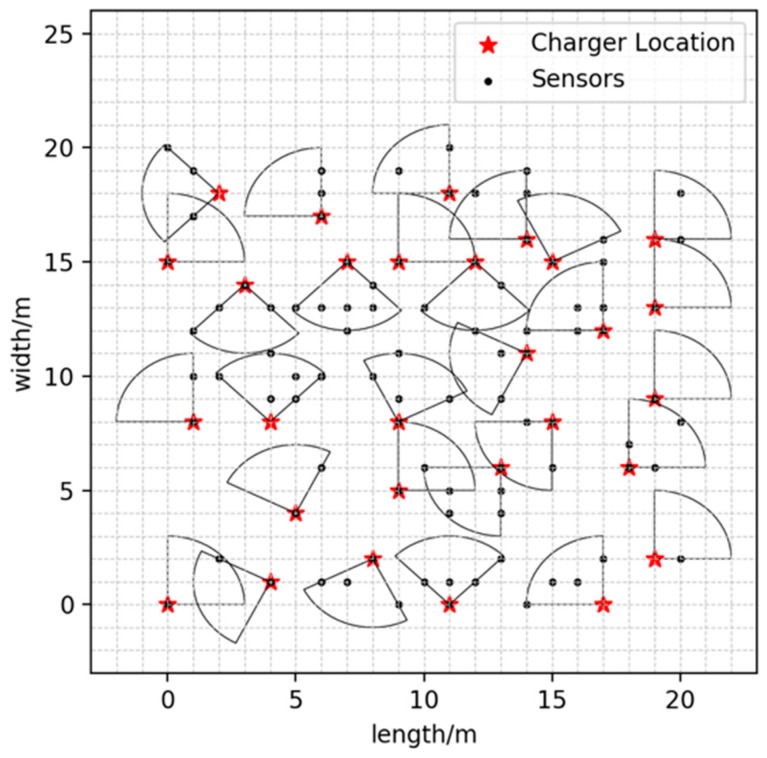
Illustration of the GMCU algorithm’s example result: DCV’s candidate charging locations and charging directions.

As shown in Figure 8, we randomly deploy 100 nodes in the 20 × 20 m^2^ area and run the GMCU algorithm to determine specified docking spots and orientations with maximum coverage utility and minimum the number of docking spots.

### 4.2. Plan Moving Path and Charging Residence Time

In this section, we plan the DCV’s charging moving path to travel through all candidate docking spots chosen by the GMCU algorithm and the charging residence times at each docking spot to maintain the network’s continuous working and optimize the overall charging energy efficiency.

**Figure 9:**
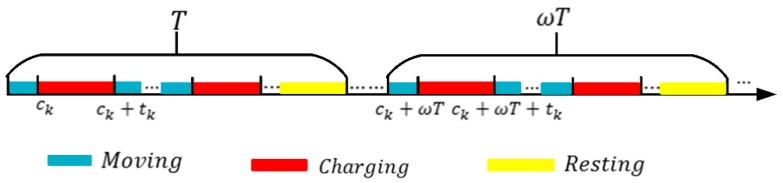
Periodic behavior of the charging car.

Firstly, we introduce the charging cycle T. As shown in Figure 9, the charging cycle T consists of the DCV’s moving time, the charging residence time at each docking spot, and the rest time at the base station. The moving time is determined by the length of charging path. The charging residence time at each docking spot is determined by charging energy requirement of sensors covered by the DCV.

To achieve the goal of maintaining network perpetually, the charging process can be repeated periodically. Then this periodical charging cycle must meet two requirements:

(1) The energy received by a sensor is greater or equal to the energy consumed in a charging cycle;
(2) The residual energy value of a node will not be lower than $E_{min}$ during a charging cycle.

The cover sets charged by the DCV at dock spot $s_{k}$ on charging orientations $\vec{\theta_{s_{k}}^{l}}$: SNC$\left(s_{k},\vec{\theta_{s_{k}}^{l}}\right)$=$\left\{o_{1}^{k_{l}},o_{2}^{k_{l}},\cdots ,o_{m}^{k_{l}}\right\}$. We can derive the minimal charging residence time $t_{k}$ according to the charging cover sets $SNC\left(s_{k},\vec{\theta_{s_{k}}^{l}}\right)$ at docking spot $s_{k}$:

(22) $$t_{k}=\;\underset{o_{i}\in SNC\left(s_{k},\vec{\theta_{s_{k}}^{l}}\right)}{\max}\left\{\frac{\omega_{o_{i}}}{P_{k,i}\left(s_{k},o_{i}\right)}\right\}\times T$$

Here $\omega_{o_{i}}$ denotes the energy consumption of sensor node $o_{i}$, $P_{k,i}\left(s_{k},o_{i}\right)$ denotes the receiving power of the sensor node $o_{i}$ when the DCV is at docking spot $s_{k}$.

We denote $c_{k}$ as the arrival time of the DCV at docking spot k in the first cycle. Denote $d_{0,1}$ as the distance between the base station and the first docking spot, $d_{l,l+1}$ as the distance between lth and (l+1) th docking spot.

(23) $$c_{k}=\overset{k-1}{\underset{l=0}{\sum }}\frac{d_{l,l+1}}{v}+\;\overset{k-1}{\underset{l=1}{\sum }}t_{l}$$

Here $t_{l}$ denotes the DCV’s charging time at docking spot $s_{l}$.

According to Figure 5, we can derive from Equation (22):

(24) $$E_{max}-\underset{o_{i}\in SNC\left(s_{k},\vec{\theta_{s_{k}}^{l}}\right)}{\min}\left\{e_{o_{i}}\left(c_{k}\right)\right\}\geq E_{max}-\underset{o_{i}\in SNC\left(s_{k},\vec{\theta_{s_{k}}^{l}}\right)}{\min}\left\{e_{o_{i}}\left(T\right)\right\}$$

That is to say $e_{o_{i}}\left(m\times T+c_{k}\right)$ is the minimum value of $e_{o_{i}}\left(\tau \right)$. To have $e_{o_{i}}\left(\tau \right)\geq E_{min}$, it is sufficient to have:

(25) $$e_{o_{i}}\left(m\times T+c_{k}\right)=e_{o_{i}}\left(m\times T\right)-c_{k}\times \omega_{o_{i}}\geq E_{min},o_{i}\in SNC\left(s_{k},\vec{\theta_{s_{k}}^{l}}\right)$$

while m≥1:

(26) $$e_{o_{i}}\left(m\times T+c_{k}\right)=e_{o_{i}}\left(m\times T\right)-c_{k}\times \omega_{o_{i}}\;=e_{o_{i}}\left(\left(m-1\right)\times T+c_{k}+t_{k}\right)-\left\{m\times T-\left[\left(m-1\right)\times T+c_{k}+t_{k}\right]\right\}-c_{k}\times \omega_{o_{i}}\;=e_{o_{i}}\left(\left(m-1\right)\times T+c_{k}+t_{k}\right)-\left(T-t_{k}\right)\times \omega_{o_{i}}\;=E_{max}-\left(T-t_{k}\right)\times \omega_{o_{i}}$$

Therefore, if Equation (27) holds, we have $e_{o_{i}}\geq E_{min}$, the sensor $s_{k}$ can working continuously.

(27) $$E_{max}-\left(T-t_{k}\right)\times \omega_{o_{i}}\geq E_{min}$$

We can get the Charging Cycle T when the two periodical charging requirements are met. Then we plan the DCV’s charging moving path. 

When the DCV moves along the shortest Hamiltonian circle, we can achieve the maximum energy efficiency η. 

We can proof this based on contradiction. Suppose the shortest travel route for the Hamilton Circle is $L=\left\{s_{1}⇢s_{2}⇢\cdots ⇢s_{M}\right\}$, and there exists a move route L^={s3⇢s2⇢⋯⇢sM⇢s1}. Assume that η^≥η is established.

(28) η^=EEDCV^=∑k=1M∑i=1NPk,i(sk,oi)×tk^Pout×∑k=1Mtj+ωc×L^

(29) η=EEDCV=∑k=1M∑i=1NPk,i(sk,oi)×tkPout×∑k=1Mtk+ωc×L

The energy received by the node in this cycle is equal to the energy consumed. The numerator of Equations (28) and (29) are equal. Because L≤L^, η^≤η, thus leading to a contradiction. Therefore, we can dispatch the DCV moving along the shortest Hamiltonian circle to achieve the maximum energy efficiency.

We redefine Equation (11) as Equation (30)

(30) $$\begin{array}{cc} max & \eta \end{array}\;\begin{array}{cc} s.t. & \;t_{k}=\;\underset{o_{i}\in SNC\left(s_{k},\vec{\theta_{s_{k}}^{l}}\right)}{\max}\left\{\frac{\omega_{o_{i}}}{P_{k,i}\left(s_{k},o_{i}\right)}\right\}\times T \end{array}\;E_{max}-\left(T-t_{k}\right)\times \omega_{o_{i}}\geq E_{min}\;\;\;T=T_{res}+T_{TSP}+\overset{M}{\underset{k=1}{\sum }}t_{k}\;\;P_{out}\overset{M}{\underset{k=1}{\sum }}t_{k}+\omega_{c}\times L_{c}\leq C_{max}$$

Here η denotes Energy Charging Efficiency, $\omega_{o_{i}}$ denotes the energy consumption of sensor node $o_{i}$, $P_{k,i}\left(s_{k},o_{i}\right)$ denotes the receiving power of the sensor node $o_{i}$ when the DCV is at docking spot $s_{k}$, $t_{k}$ denotes the DCV’s charging time at docking spot $s_{k}$, $T_{res}$ denotes rest time of the DCV, $T_{TSP}$. denotes the moving time of the DCV, the sum of $t_{k}$ denotes the total charging time of the DCV.

Finally, we get the charging residence time at each docking spot and energy efficiency by solving the planning problem.

## 5. Analysis of the DCV’s Service Capability

We use only one DCV with energy capacity of $E_{max}$ to maintain WRSN perpetually. Therefore, the network size and area size are limited. This section will specifically analyze the service capability of the DCV.

Assume that the number of stops is M, the charging time of each stop is $t_{k}$, the distance between adjacent stops is $d_{k-1,k}$, the length of the return route is $d_{back}$. Two constraints must be satisfied for each round of charging: (1) the energy received by each node is not less than the energy consumed, formulated as Equation (31); and (2) the DCV should not run out of energy in a round, formulated as Equation (32).

(31) $$\underset{o_{i}\in SNC\left(s_{k},\vec{\theta_{s_{k}}^{l}}\right)}{min}\left\{P_{k,i}\left(s_{k},o_{i}\right)\right\}\times t_{k}\geq \underset{o_{i}\in SNC\left(s_{k},\vec{\theta_{s_{k}}^{l}}\right)}{max}\left\{\omega_{o_{i}}\right\}\times \;\left(\left(t_{1}+\cdots +t_{M}\right)+\frac{d_{1,2}+\cdots +d_{M-1,M}+d_{back}}{v}\right),0\leq k\leq M$$

(32) $$P_{out}\times \left(t_{1}+\cdots +t_{k}+\cdots +t_{M}\right)+\left(d_{1,2}+d_{2,3}+\cdots +d_{M-1,M}+d_{back}\right)\times \omega_{c}\leq C_{max}$$

Here,$\;P_{out}$ is the charger’s transmission power, v denotes the moving speed of the DCV,$\;\omega_{c}$ denotes DCV’s consumption power of moving, $C_{max}$ denotes maximum energy capacity of the DCV.

We first analyze the maximum size of area. Assuming that there are only two nodes in the network and they are on the diagonal line of the network, the consuming power is the minimum $\omega_{min}$, the DCV stops at the nodes respectively, and the receiving power of the nodes is both $P_{out}$. Then the number of stops is two (M=2), the shortest distance of moving route is $2\sqrt{2}*l$, l denotes length of the network ,then we can get Equation (33)

(33) $$l_{max}=\frac{C_{max}\times \left(P_{out}-2\times \omega_{min}\right)}{2\sqrt{2}\times \left(\frac{P_{out}\times \omega_{min}}{v}+\omega_{c}\times \left(P_{out}-2\times \omega_{min}\right)\right)}$$

Secondly, we analyze the minimum size of area. Assume that the nodes are evenly distributed in the network, the consuming power is the maximum $\omega_{max}$, the DCV stops at the nodes respectively, and the receiving power is all the minimum $P_{min}$. Then the number of stops is formulated as Equation (34).

(34) ⌈l22×D⌉2

The longest distance of move route is formulated as Equation (35).

(35) $$2\times \left(M-1\right)\times \sqrt{2}\times D$$

We bring Equations (34) and (35) into Equations (31) and (32) to get Equations (36).

(36) $$M=\frac{-b\pm \sqrt{b^{2}-4\times a\times c}}{2\times a}\;\{\begin{array}{c} a=2\sqrt{2}\times \omega_{max}\times D\times P_{min}\\ \begin{array}{c} b=2\sqrt{2}\times D\times \omega_{c}\times v\times \left(P_{min}-\omega_{max}\right)\\ -2\sqrt{2}\times \omega_{max}\times D\times P_{min} \end{array}\\ c=v\times \left(P_{min}-\omega_{max}\right)\times \left(C_{max}+2\sqrt{2}\times D\times \omega_{c}\right) \end{array}$$

Therefore, the minimum length of area is $L_{min}$, formulated as Equation (37).

(37) $$L_{min}=\lfloor \sqrt{d^{2}\times M}\rfloor$$

When the network area is the smallest, assuming that the charger can charge CN nodes simultaneously at most, the number of nodes can reach the maximum. Then the maximum number of nodes is CNS, formulated as Equation (38).

(38) CNS=M×CN

In summary, when the size of the area is between $L_{min}$ and $L_{max}$ and the size of network is less than CNS, the proposed charging model and approximate algorithm can satisfy the two constraints: 1) the energy received by each node is not less than the energy consumed; and 2) the DCV should not run out of energy in a round.

## 6. Simulation Experiments

In this section, we describe comprehensive simulation experiments to investigate the algorithms’ performance under different influence factors, such as grid size, area size, and network size. In the existing literature, there are no related works that study mobile directional charging problem in WRSN. Therefore, we conducted simulations experiments and compared charging efficiency with mobile omnidirectional charging models [14]. The simulation experiments were performed on a 64-bit Windows 10 system; the programming languages were C++ and Python. The algorithms were realized in the C++ language. Additionally, the visualization of deployment results was realized in Python. In the simulation experiments, we set up the parameters of the DCV and rechargeable sensor network, as in Table 4.

### 6.1. Comparison Experiments on Different Grid Size

**Figure 10:**
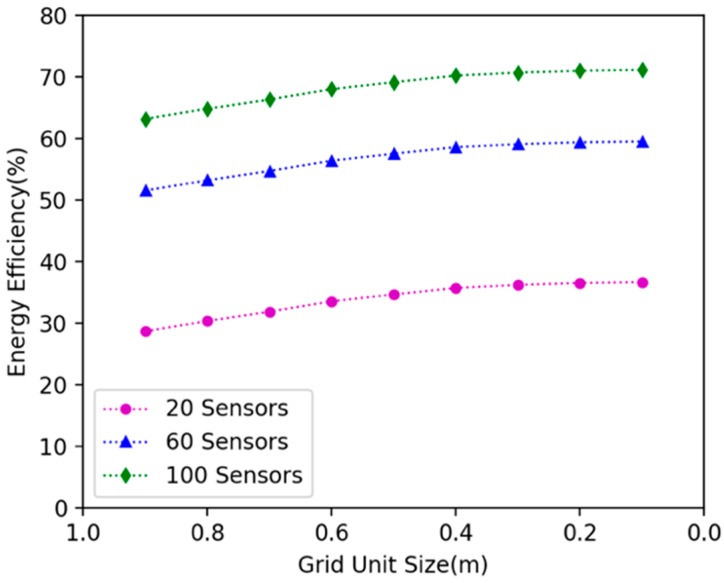
The effect of different grid sizes on the energy efficiency.

**Figure 11:**
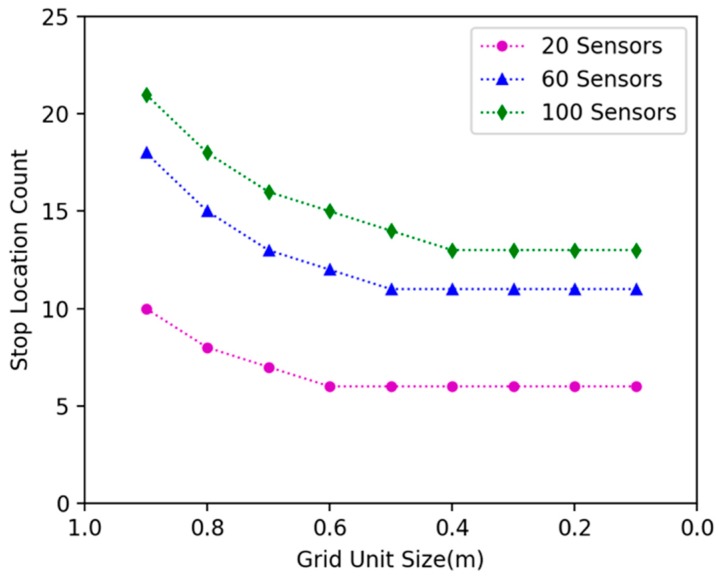
The effect of different grid sizes on the number of specified docking spots.

In our approach, we discretized the continuous 2D plane with gridding. We investigated how grid size affects the algorithm’s performance. We randomly deployed 20, 40 and 60 nodes in the 15 × 15 m^2^ area, changed the grid size, and explored the variation of energy efficiency and docking spots number. Figure 10 shows that with the decrease of grid size, the energy efficiency of the DCV increase. Additionally, a stable grid size tends to be 0.2 m. Figure 11 shows that with the decrease of grid size, the number of specified docking spots decreases. Additionally, it tends to be stable when grid size is .2 m. 

### 6.2. Comparison Experiments on Different Network Size And Area Size

**Figure 12:**
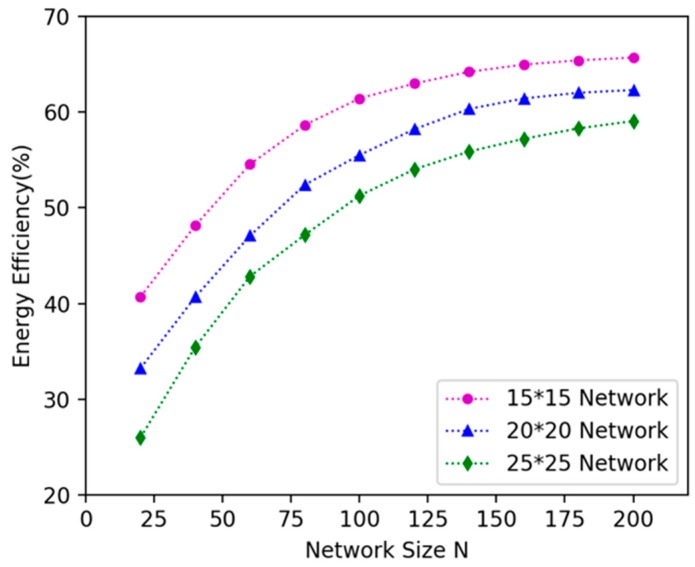
The influence of network size N on energy efficiency of the DCV.

**Figure 13:**
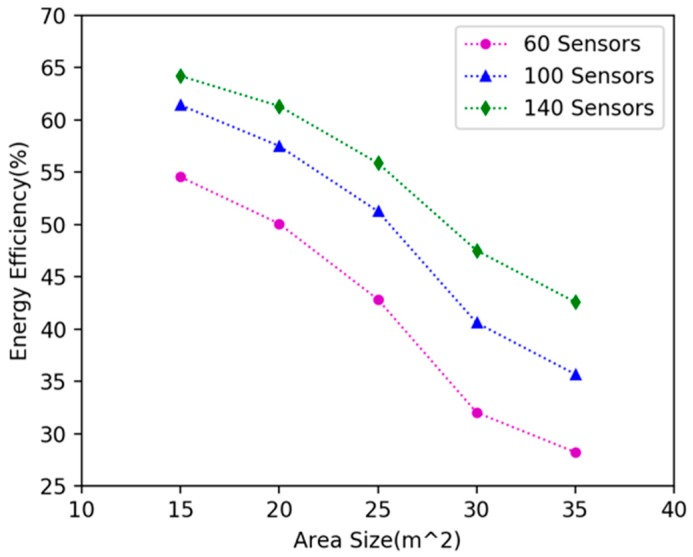
The influence of area size on energy efficiency of the DCV.

We investigated how network size affects the algorithm’s performance. We randomly deployed 20, 40, 60, 80, 100, 120, 140, 160, 180, 200 nodes in 15 × 15 m^2^, 20 × 20 m^2^, 25 × 25 m^2^, 30 × 30 m^2^, 35 × 35 m^2^ plane areas respectively, and explored the variation in the energy efficiency of DCV. It can be seen in Figure 12 that as the number of nodes increases, the energy efficiency increases; because the number of nodes increases in the same area, the number of nodes can be covered by the DCV increases, so more energy is received by the nodes, and the energy efficiency is improved. As shown in Figure 13, when the number of nodes remains unchanged and the area becomes larger, the energy efficiency decreases. This is because as the area becomes larger, the distance between nodes becomes larger, the moving path of the DCV becomes longer, and the energy consumed on moving increases, which leads to the decrease of the energy efficiency of the DCV.

### 6.3. Comparison Experiments on Mobile Omnidirectional and Directional Charging

**Figure 14:**
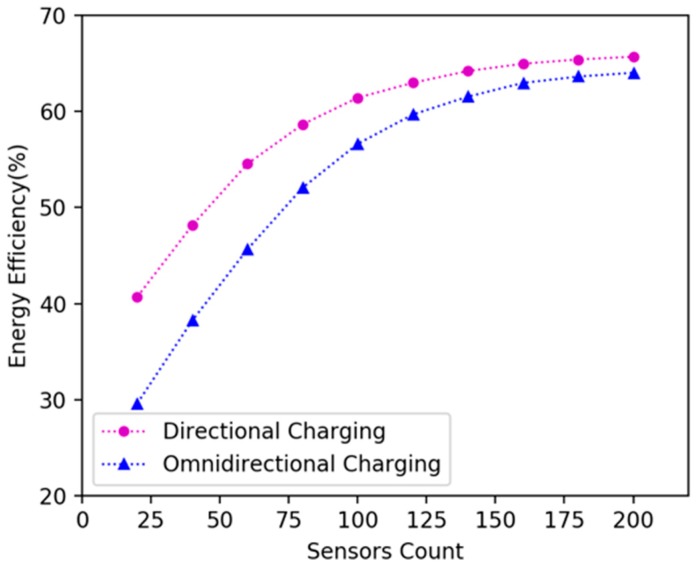
Energy efficiency of the DCV and omnidirectional charging vehicle in 15 × 15 m^2^ area.

**Figure 15:**
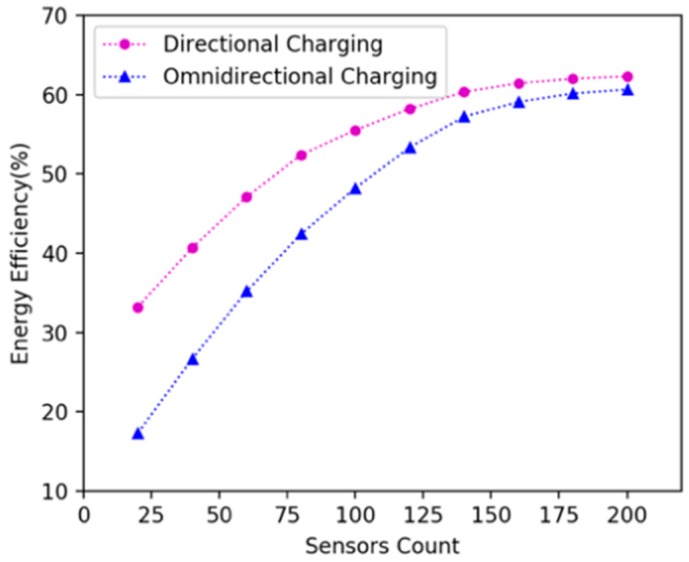
Energy efficiency of the DCV and omnidirectional charging vehicle in 20 × 20 m^2^ area.

**Figure 16:**
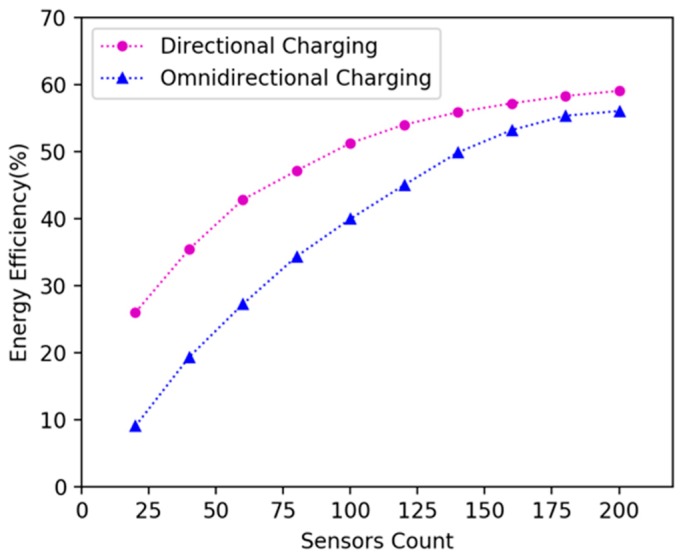
Energy efficiency of the DCV and omnidirectional charging vehicle in 25 × 25 m^2^ area.

In the existing literature, there are no related works that use directional charging model for mobile charging in WRSN. Therefore, we conducted simulation experiments and compared charging efficiency with mobile omnidirectional charging [14]. We randomly deployed 20, 40, 60, 80, 100, 120, 140, 160, 180 and 200 nodes in 15 × 15 m^2^, 20 × 20 m^2^ and 25 × 25 m^2^ areas. In experiments, we used DCV and omnidirectional charging vehicle respectively to charge the network according to the algorithms proposed in this paper, and compare their energy efficiency. Figure 14, Figure 15 and Figure 16 show the variation of energy efficiency in different area size and network size. The experiments show that the energy efficiency of DCV is higher than that of omnidirectional charging vehicle in the network with sparse nodes. As the node density increases, the energy efficiency of DCV and omnidirectional charging vehicle will gradually converge. Hence our mobile directional charging algorithm is more suitable in a network with sparse nodes compared with mobile omnidirectional charging.

## 7. Conclusions

In this paper, we investigated the DCV’s charging efficiency optimization problem in RWSN while maintaining sensor network working continuously. We proved that the problem is NP-hard. Firstly, we proposed the coverage utility of directional charging. Then we transformed the finding of candidate docking spots and their charging directions on the 2D plane into a two-objective optimization problem of minimizing number of stop points and maximizing charging coverage utility. Additionally, we proposed a greedy approximation algorithm to solve the two-objective optimization problem and find the set of candidate stop points of the DCV. Finally, we planned the DCV’s charging moving path to travel through all candidate docking spots to maintain the network’s continuous working and optimize the overall energy charging efficiency. We theoretically analyzeed the DCV’s charging service capability, and performed the comprehensive simulation experiments. The simulation experiment results show that energy charging efficiency is higher than omnidirectional charging model in the sparse networks.

As stated in the literature [40], WPT has several limitations when applied to a WSN. First, it has very low energy transfer efficiency as distance increases. Second, it is sensitive to obstruction between an energy source and a receiver. Therefore, this technology is only suitable in the ultra-low-power WSN scenario. In future work, we will further investigate more practical energy replenishment optimization problem in WSN, in which we can use a hybrid energy replenishing scheme, such as wireless charging for ultra-low-power sensor nodes and solar energy harvesting for high-power sensor nodes in the network.

## Figures and Tables

**Table 1:** Symbol and Notations.

Symbol	Meaning
$s_{k}$	Coordinate of docking spot k
$o_{i}$	Coordinate of sensor node i
$\vec{\theta_{s_{k}}}$	DCV’s charging orientation at docking spot k
$d\left(s_{k},o_{i}\right)$	Euclidean distance between sensor node $o_{i}$ and the docking spot $s_{k}$
$P_{k,i}\left(s_{k},o_{i}\right)$	DCV’s energy transfer function at docking spot $s_{k}$ for sensor node $o_{i}$
A	Charging angle of DCV (°)
v	The moving speed of DCV (m/s)
D	Effective charging distance of DCV (m)
$P_{out}$	Energy transmit power of DCV (J/m)
$\omega_{c}$	Moving energy consumption of DCV (J/m)
$C_{max}$	Energy capacity of DCV
$\omega_{o_{i}}$	Energy consumption of sensor node i
$e^{s}$	Energy consumption for sensing one unit data
$e^{t}$	Energy consumption for transmitting one unit data
$e^{r}$	Energy consumption for receiving one unit data
$R_{o_{i}}$	Sensing data generation rate of sensor node i
L × L	Size of the area

**Table 2:** The Procedure of the GMCU (Greedy approximation algorithm of Maximum Coverage Utility) Algorithm.

**GMCU algorithm: find candidate docking spots and their charging directions**
**Input**: The length of area: *L*; Farthest distance DCV can reach: *D*; Charging angle of DCV: *A* Discrete the *L* x *L* plane into grids, get the vertex set of grids: $CS=\left\{cds_{1},cds_{2},\cdots ,cds_{k},\cdots ,cds_{num}\right\}$,$\;cds_{k}$ is coordinates of vertexes DDS=∅, SANC=∅, k=0 //DDS candidate docking spots //SANC set of cover set which associated with *DDS* **While**O≠∅ // *O* set of sensor nodes $SNC_{temp}=\emptyset$, $U_{temp}=0$, $CDS_{temp}=0$ //find a stop point with max cover utility **While** k<len(CS) **Call** DMCU($cds_{k}$) to get max coverage utility $U_{max}\left(cds_{k}\right)$, cover set $SNC_{k}$ at docking point $cds_{k}$ with charging direction $\vec{\theta_{s_{k}}}$ **If** $U_{max}\left(cds_{k}\right)>U_{temp}$ $\;\;U_{temp}=U_{max}\left(cds_{k}\right)$ $\;\;SNC_{temp}=SNC_{k}$ $\;\;CDS_{temp}=cds_{k}\;$ **End If** k=k+1 **End While** $SANC=SANC\cup^{\;}\{SNC_{temp}\}$ $DDS=\;DDS\cup^{\;}\{CDS_{temp}\}$ $O=O-SNC_{temp}$ *CS = CS* – {$CDS_{temp}\}$ *k = 0* **End While** **Output**: set of docking points DDS and set of charging cover sets SANC at related charging directions

**Table 3:** The Procedure of the DMCU Algorithm.

**DMCU algorithm: Find the max utility, cover set and charging orientation at** $s_{k}$
**Input:** Sensor node set: $O=\left\{o_{1},o_{2},\cdots ,o_{i},\cdots ,o_{N}\right\}$; Coordinates of certain docking spot $s_{k}$: $\left(c_{x},c_{y}\right)$; Farthest charging distance DCV can reach: *D*; Charging angle of DCV: *A* OCS=∅ // *OCS* sensors’ set possible covered by $s_{k}$ i=0 **While**i<N: //find sensors’ set *OCS* at docking spot $s_{k}$ Calculate Euclidean distance between sensor $o_{i}$ and docking spot$\;d_{i}$ **If** $d_{i}<D$: $OCS=OCS\cup^{\;}\left\{o_{i}\right\}$ **End If** i=i+1 **End While** **If**OCS≠∅: L=len(OCS) Calculate the all possible charging angles: $\varphi =\left\{\gamma_{1},\gamma_{2},\cdots ,\gamma ,\cdots ,\gamma_{L}\right\}$ Sort sensors in set OCS in ascending order according to the value of angles. DCS=∅,k=0 // calculate *L* directions’ cover sets **While** k<L m=0,$\;SNC_{tmp}=\emptyset$ **While** m<L **If** $\gamma_{k}\leq \gamma_{m}\leq \left(\gamma_{k}+A\right)\%360$: $SNC_{tmp}=SNC_{tmp}\cup^{\;}\{o_{m}\}$ **End If** *m* = *m* + 1 **End while** $DCS=DCS\cup^{\;}\{SNC_{tmp}\}$ k=k+1 **End while** $CUS_{tmp}=\emptyset ,SNC_{tmp}=\emptyset ,\;j=0$, $\gamma_{tmp}=0$ // find the cover set with max utility **While** j<len(DCS) Calculate cover utility CUS[j] of DCS[j] **If** $CUS\left[j\right]>CUS_{tmp}$ $CUS_{tmp}=CUS\left[j\right]$ $\;\;SNC_{temp}=DCS\left[j\right]$ $\gamma_{tmp}=$ φ[j] **End If** *j* = *j* +1 **End While** $U_{max}=CUS_{tmp}$, $SNC=SNC_{temp}$, $\gamma =\gamma_{tmp}$ **End If** **Output**: max utility $U_{max}$, covered set SNC, direction γ

**Table 4:** Parameter Setting.

Parameter	Value
$E_{max}$	10,000 J
$P_{ou}$	3 J/s
$\omega_{c}$	0.3 J/m
D	3 m
v	0.5 m/s
$R_{o_{i}}$	randomly generated in References [1,10] b/s
$e^{s}$	0.01 mJ/b
$e^{t}$	0.06 mJ/b
$e^{r}$	0.05 mJ/b
α,β	10
The number of sensor nodes	20, 40, 60, 80, 100, 120, 140, 160, 180,200
The size of area	15 × 15 m^2^, 20 × 20 m^2^, 25 × 25 m^2^, 30 × 30 m^2^, 35 × 35 m^2^,
